# Neural Coding of Thermal Preferences in the Nematode *Caenorhabditis elegans*

**DOI:** 10.1523/ENEURO.0414-19.2020

**Published:** 2020-06-25

**Authors:** Hironori J. Matsuyama, Ikue Mori

**Affiliations:** Neuroscience Institute, Graduate School of Science, Nagoya University, Nagoya 464-8602, Japan

**Keywords:** behavioral preference, C. elegans, emotion, phase coding, thermotaxis

## Abstract

Animals are capable to modify sensory preferences according to past experiences. Surrounded by ever-changing environments, they continue assigning a hedonic value to a sensory stimulus. It remains to be elucidated however how such alteration of sensory preference is encoded in the nervous system. Here we show that past experiences alter temporal interaction between the calcium responses of sensory neurons and their postsynaptic interneurons in the nematode *Caenorhabditis elegans*. *C. elegans* exhibits thermotaxis, in which its temperature preference is modified by the past feeding experience: well-fed animals are attracted toward their past cultivation temperature on a thermal gradient, whereas starved animals lose that attraction. By monitoring calcium responses simultaneously from both AFD thermosensory neurons and their postsynaptic AIY interneurons in well-fed and starved animals under time-varying thermal stimuli, we found that past feeding experiences alter phase shift between AFD and AIY calcium responses. Furthermore, the difference in neuronal activities between well-fed and starved animals observed here are able to explain the difference in the behavioral output on a thermal gradient between well-fed and starved animals. Although previous studies have shown that *C. elegans* executes thermotaxis by regulating amplitude or frequency of the AIY response, our results proposed a new mechanism by which thermal preference is encoded by phase shift between AFD and AIY activities. Given these observations, thermal preference is likely to be computed on synapses between AFD and AIY neurons. Such a neural strategy may enable animals to enrich information processing within defined connectivity via dynamic alterations of synaptic communication.

## Significance Statement

*Caenorhabditis elegans* can assign a hedonic value to a temperature depending on past feeding experiences. They prefer the temperature associated with food intake, whereas lose the preference to one associated with starvation. This paradigm provides a model to elucidate how the sensory preference is encoded by the nervous system. We show here that thermal preference is encoded by temporal interaction, especially phase shift, between the responses of sensory neurons and their postsynaptic interneurons. Furthermore, the neuronal activity patterns we observed here were well correlated with behavioral outputs. Although previous studies have revealed that amplitude or frequency of interneuron responses encodes temperature information, we added a new mechanism by which phase shift between sensory neurons and interneurons encode thermal preference.

## Introduction

Animals alter their preferences based on past experiences. The ability to modify the hedonic values of sensory stimuli is central to brain function, and enables animals to respond flexibly to environments. Defining neural circuits that mediate the assignment of hedonic values to sensory stimuli has been a great challenge in neuroscience. According to the previous studies, the hedonic values of sensory stimuli can be encoded by several circuit motifs (Tye, 2018), including labeled lines (Root et al., 2014; Wang et al., 2018), divergent paths (Namburi et al., 2015; Beyeler et al., 2016, 2018) and opposing components within a defined connectivity (Barbano et al., 2016; Nieh et al., 2016; Stamatakis et al., 2016; Li et al., 2018). The circuit topology is certainly important for conveying hedonic information, but temporal activity patterns thereof is also critical (Ainsworth et al., 2012). However, how the circuit logic for preference alteration is implemented by neural dynamics remains unclear.

To address this question, the nematode *Caenorhabditis elegans* provides an ideal model due to its compact nervous system and a rich repertoire of behavioral plasticity. The *C. elegans* nervous system consists of only 302 identifiable neurons, and their wiring diagram was completely identified (White et al., 1986). This knowledge of neural anatomy allows us to determine the sites of neural computations at single-cell resolution and track information flow from sensory input to terminal motor output. Despite of its compact nervous system, this small worm exhibits a wide variety of behavioral plasticity for several sensory cues, including thermal (Hedgecock and Russell, 1975), mechanical (Rankin et al., 1990), olfactory (Colbert and Bargmann, 1995), and taste cues (Saeki et al., 2001; Kunitomo et al., 2013).

*C. elegans* exhibits thermal preference behavior called thermotaxis, and its thermal preference is modified by past feeding experiences. When animals are cultivated at a certain temperature with food, they are attracted to the cultivation temperature on a thermal gradient. By contrast, animals cultivated in the absence of food at the same temperature lose the attraction to the cultivation temperature (Hedgecock and Russell, 1975; Mohri et al., 2005; Kodama et al., 2006; Nishio et al., 2012). Given this behavioral switching, worms are considered to recognize temperature associated with food-intake as an attractive cue, whereas temperature associated with starvation as non-attractive cue. This themotaxis can be a model paradigm to elucidate the fundamental circuit logic for the preference alteration of animals, because the thermotaxis is known to be regulated by a simple neural circuit. Neuronal ablation experiment have proposed a simple neural circuit for the thermotaxis, consisting of sensory neurons (AFD, AWC), interneurons (AIY, AIZ, RIA), and motor neurons (SMD, RMD; Mori and Ohshima, 1995; Kuhara et al., 2008). Physiologic approaches have documented that primary sensory neurons AFD respond to thermal stimuli (Kimura et al., 2004; Clark et al., 2006; Ramot et al., 2008), and the lower temperature threshold at which AFD neurons start to respond is shifted depending on the past cultivation temperature (Kobayashi et al., 2016).

According to the previous studies, the AFD response property is not altered by starvation (Kodama et al., 2006; Tsukada et al., 2016). Furthermore, the previous genetic studies for mutants defective in associative learning between temperature and feeding experiences have revealed that insulin-like signaling pathway and calcium-activated phosphatase function in interneurons involved in thermotaxis (Mohri et al., 2005; Kodama et al., 2006; Kuhara and Mori, 2006). Given these observations, starvation is likely to modulate the interaction between AFD thermosensory neurons and downstream interneurons AIY, AIZ, and RIA, thereby allowing starved animals to reduce attraction to the cultivation temperature. Especially, AFD-AIY interaction appears to be critical, because AFD directly project onto AIY via chemical synapses (White et al., 1986). However, how the starvation experience modifies neural communication between AFD and AIY neurons has not been investigated.

The present study is devoted to investigate the relationship between AFD-AIY interaction and starvation-induced preference alteration in thermotaxis. Based on simultaneous AFD-AIY calcium imaging and quantitative behavioral analysis, we report here that starvation alters temporal interaction between AFD and AIY, thereby allowing starved animals to switch their behavioral preferences. In particular, we show that time lag or phase shift of AFD-AIY communication encodes thermal preferences. In addition to the knowledge that the amplitude or frequency of AFD-driven AIY responses encodes thermotaxis (Kuhara et al., 2011; Hawk et al., 2018; Goodman and Sengupta, 2019), our findings should shed light on phase encoding of thermal information by AFD and AIY, suggesting that sensory preference is encoded by the alteration of temporal communication within defined connectivity.

## Materials and Methods

### *C. elegans* strains

*C. elegans* strains were maintained by standard protocols by Brenner (1974). *C. elegans* strains were cultivated on nematode growth medium (NGM) plates seeded with an *Escherichia coli* OP50 strain [*Caenorhabditis* Genetics Center (CGC)]. N2 (Bristol) was used as the wild-type strain. Transgenic strain for Ca^2+^ imaging was derived from N2 by injecting plasmid DNA into hermaphrodite gonad. The strain used in Ca^2+^ imaging is IK2628 *njEx1062[gcy-8p::R-CaMP2, AIYp::GCaMP3, ges-1p::nls-TagRFP]*. *gcy-8p-synthetic intron::R-CaMP2::unc-54 3’UTR* (50 ng/μl), *AIYp::GCaMP3* (50 ng/μl), and *ges-1p::nls-TagRFP* (70 ng/μl) were co-injected. Adult hermaphrodites were used in all experiments. All animal procedures were performed in accordance with the Nagoya University animal care committee’s regulations.

### Behavioral assays

Thermotaxis assays were performed as described previously (Ito et al., 2006). Ten adult animals were placed on NGM plate with food (OP50), allowed to lay eggs for 2.5 h at room temperature, and removed. The eggs were cultivated at 20°C for 69 h. Grown adult animals were collected and washed twice with NG buffer (0.3% NaCl, 1 mm CaCl_2_, 1 mm MgSO_4_, and 25 mm potassium phosphate, pH 6.0) to remove food attached to their bodies, and placed onto NGM plate with or without food, and conditioned at 20°C for 3 h. These animals were defined as well-fed and starved animals. Thermotaxis assays were conducted on agar plate with linear thermal gradient. Approximately 50–120 conditioned animals were collected and washed twice with NG buffer, placed at the center of thermal gradient without food, and allowed to freely move for 1 h.

### Behavioral recording

The recordings of behavior were performed by multi-worm tracker (MWT; Swierczek et al., 2011) with a CMOS sensor Camera Link Camera (8-bit, 4096 × 3072 pixel; CSC12M25BMP19-01B, Toshiba-Teli), a lens adaptor (F-TAR2), a Line-Scan Lens (35 mm, f/2.8; YF3528, PENTAX), and a PCIe-1433 camera-link frame grabber (781169-01, National Instruments). The camera was located above the assay plate so that an image pixel is equal to 33.2 μm. The frame rate of the recording was ∼13.5 Hz. The images were captured and processed by software package written in LabView (National Instruments; Swierczek et al., 2011) and image processing library written in C++ (Swierczek et al., 2011), which detect animals and measure the positions and the postures of animals.

### Behavioral analysis

The MWT detects animals and measure the positions of their centroids and 11 segments along their bodies (Swierczek et al., 2011). Based on these data, the quantitative analysis was performed by MATLAB (MathWorks) scripts (Ikeda et al., 2018; Yamaguchi et al., 2018; Nakano et al., 2020). Several behavioral components were categorized and their frequencies were calculated by the script as follows. First, we took two-dimensional coordinate system on the assay plate where *x*-axis is parallel to thermal gradient, and *y*-axis is parallel to isothermal line. For each frame, moving vector ***r***(*t*) of each animal was defined as a displacement vector from the previous centroid 1 s before to the current centroid. Moving direction θ was calculated as the angle of moving vector ***r*** from *x*-axis. θ is ranged from 0° (warmer direction) to 180° (colder direction). “Curving rate” was quantified by the angle ϕ between the current moving vector ***r***(*t*) and the previous one 1 s before ***r***(*t*-1). “Omega turn,” a large turning behavior in which an animal’s head curls back touching the tail with Ω-shaped posture, was detected by an underestimation of body length in the MWT system. When an animal exhibits Ω-turn, the body length is clearly decreased in the system. Accordingly, if the body length was estimated smaller than 1.5 SD from the mean and curving rate was larger than 90°/s, we regarded the animal as exhibiting Ω turn. “Reversal” was detected if mean curving angle ϕ within three frames was >150°/s.

### Calcium imaging

**Figure 1.**
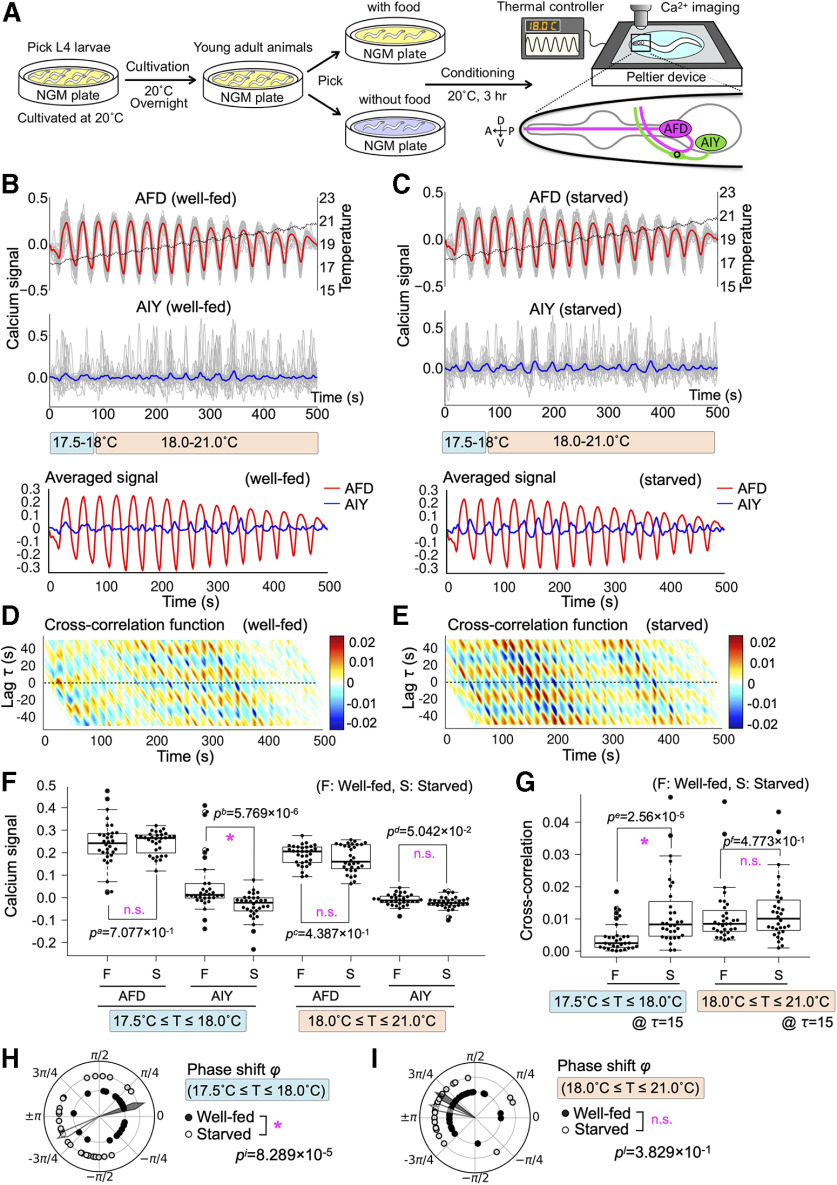
AFD-AIY temporal interaction in response to oscillatory thermal ramp from 17.5°C to 21.0°C (*Osci1721*) was altered by starvation. ***A***, The schematics of simultaneous calcium imaging of AFD and AIY under time-varying thermal stimuli. Well-fed and starved animals were conditioned at 20°C for 3 h with and without food, respectively. Genetically encoded calcium indicators R-CaMP2 and GCaMP3 were expressed in AFD and AIY, respectively. Calcium signals were monitored from AFD cell bodies and AIY axons (shown by a circle on schematic AIY figure). ***B***, AFD-AIY calcium responses of well-fed animals under thermal stimuli *Osci1721* (*n* = 32). Dashed black line indicates mean of the thermal stimuli. The gray lines indicate individual traces of calcium signals of AFD and AIY. The red and blue lines represent the mean of individual AFD and AIY signals, respectively. The blue and red bars indicate the temperature range of lower (17.5–18.0°C) and higher one (18.0–21.0°C). ***C***, AFD-AIY calcium responses of starved animals under thermal stimuli *Osci1721* (*n* = 32). ***D***, Cross-correlation function between AFD and AIY activities for well-fed animals; *x*-axis and *y*-axis represent time and time lag, respectively. Color map indicates value of the cross-correlation function. ***E***, Cross-correlation function between AFD and AIY activities for starved animals. ***F***, Differences in AFD and AIY calcium signals between well-fed and starved animals. Four datasets on the left: the results at lower temperature (17.5–18.0°C). The peak values of AFD signals and the values of AIY signals when AFD signals reach their peaks. Four datasets on the right: the results at higher temperature (18.0–21.0°C). Differences in AFD and AIY signals between well-fed and starved animals were tested for statistical significance by using Brunner–Munzel test; *p* values <0.05/5 = 0.01 (1%) were considered statistically significant, because a dataset was tested five times: (1) difference in calcium signals, (2) difference in cross-correlation, (3) difference in phase shift distribution between well-fed and starved animals, (4) normality of phase shift distribution, and (5) bias of phase shift distribution. Asterisk indicates *p *<* *0.01 (statistically significant), and n.s. indicates *p *≥* *0.01 (not significant). ***G***, Difference in the cross-correlation function between well-fed and starved animals. Brunner–Munzel test was used for statistical analysis; *p* values <0.05/5 = 0.01 (1%) were considered statistically significant. Asterisk indicates *p *<* *0.01 (statistically significant), and n.s. indicates *p *≥* *0.01 (not significant). ***H***, Difference in phase shift between AFD and AIY at lower temperature (17.5–18.0°C). The black dots indicate phase shift between AFD and AIY responses for well-fed animals, and white dots are for starved animals. The black and white arrowheads represent mean directions of individual phase shift vectors. Mardia–Watson–Wheeler test was used for statistical analysis; *p* values <0.05/5 = 0.01 (1%) were considered statistically significant. Asterisk indicates *p *<* *0.01 (statistically significant), and n.s. indicates *p *≥* *0.01 (not significant). ***I***, Difference in phase shift between AFD and AIY at higher temperature (18.0–21.0°C). Quantifications and statistical tests were done in the same manner as ***H***.

Calcium imaging was performed based on the previous study (Kobayashi et al., 2016). Animals expressing R-CaMP2 and GCaMP3 in AFD and AIY respectively were cultivated at 20°C. Young adult animals were placed onto a NGM plate with food or without food, and conditioned at 20°C for 3 h. A well-fed or starved animal was placed on a 10% agarose pad with polystyrene beads for immobilization, and the animal was covered by a coverslip. Thermal stimuli were delivered to the animal, and the fluorescence intensities of AFD cell body and AIY axon (shown by a circle on a schematic figure of AIY in Fig. 1*A*), were captured. Image processing was performed by MetaMorph (Molecular Devices).

### Analysis of calcium signals

The fluorescence intensities *F*(*t*) of R-CaMP2 and GCaMP3 were rescaled as $y\left(t\right)=\Delta F\left(t\right)/F_{0}=\left[F\left(t\right)-F_{0}\right]/F_{0}$ where $F_{0}$ is the minimum value of *F*(*t*) within an animal. *y*(*t*) of AFD and AIY were normalized between 0 and 1, and were detrended by Butterworth filter to remove long-term changes of the signals. The detrended signals of AFD and AIY were named as $y_{\text{AFD}}$ and $y_{\text{AIY}}$, and we called them as simply “calcium signals” in the present paper.

To quantify the interaction between AFD and AIY signals, cross-correlation as a function of both time and time displacement was calculated under non-stationary condition as follows:

$$C\left(t,\tau \right)=y_{\text{AFD}}\left(t\right)y_{\text{AIY}}\left(t+\tau \right),$$

where *τ* is the time displacement of AIY signal against AFD signal, and <⋅⋅⋅> indicates ensemble average. For statistical tests, we extracted peak values of the cross-correlation function as follows. First, we defined a value of time lag *τ* = τ*, and obtained the time series of cross-correlation function at that time lag as C (*t*, τ*). We extracted the values of local maxima of the function and took time average of them. The peak values were calculated for all the animals, and the statistical analyses were performed.

To quantify the phase shift between AFD and AIY responses, Hilbert transform was applied to the derivatives of AFD and AIY activities. Before applying Hilbert transform, we filtered the derivatives by Butterworth filter where passband edge frequency is 0.12 Hz and stopband edge frequency is 1.2 Hz. Filtered derivatives of AFD and AIY were named as $x_{\text{AFD}}$ and $x_{\text{AIY}}$. $x_{\text{AFD}}$ and $x_{\text{AIY}}$ represent real parts of the signals. The Hilbert transform provides imaginary parts of the signals as follows:

$${\hat{x}}_{\text{AFD}}\left(t\right)=\frac{1}{\pi }\overset{\infty }{\underset{-\infty }{\int }}\frac{x_{\text{AFD}}\left(t\prime \right)}{t-t\prime }dt\prime$$

$${\hat{x}}_{\text{AIY}}\left(t\right)=\frac{1}{\pi }\overset{\infty }{\underset{-\infty }{\int }}\frac{x_{\text{AIY}}\left(t\prime \right)}{t-t\prime }dt\prime .$$

By combining real and imaginary parts of the signals, we obtained analytic signals in complex form as follows:

$$z_{\text{AFD}}\left(t\right)=x_{\text{AFD}}\left(t\right)+i{\hat{x}}_{\text{AFD}}\left(t\right)$$

$$z_{\text{AIY}}\left(t\right)=x_{\text{AIY}}\left(t\right)+i{\hat{x}}_{\text{AIY}}\left(t\right),$$

where $i=\sqrt{-1}$. The instantaneous phases of AFD and AIY were calculated as arguments of analytic signals:

$$\theta_{\text{AFD}}\left(t\right)=\text{arg}\left[z_{\text{AFD}}\left(t\right)\right]$$

$$\theta_{\text{AIY}}\left(t\right)=\arg[z_{\text{AIY}}\left(t\right)].$$

Finally, the instantaneous phase shifts between AFD and AIY responses were obtained:

$$\varphi \left(t\right)=\theta_{\text{AFD}}\left(t\right)-\theta_{\text{AIY}}\left(t\right),$$

*φ* was rescaled such that −π≤φ≤π. When AFD and AIY derivatives showed in-phase synchronization, *φ* = 0, whereas when they showed anti-phase synchronization, *φ* = *±π.* If AFD response was followed by AIY response, *φ* > 0, whereas if AFD response was behind AIY response, *φ* < 0. For statistical tests, we extracted the values of phase shift *φ*(*t*) when AIY derivatives reach peaks. We calculated weighted mean directions of these phase shift vectors as follows:

$${\bar{\varphi }}_{j}=\text{arg}\left[\overset{N}{\underset{k=1}{\sum }}R_{jk}\text{exp}\left(i\varphi_{jk}\right)\right],$$

where *j* is a suffix for representing individuals, *k* is a suffix for representing events where AIY derivatives reach peaks, *N* is the total number of the events, and $i=\sqrt{-1}$. Not to overestimate the phase shifts sampled when the derivatives of AIY reach peaks with small amplitude, the amplitude of AIY derivatives *R_j_* is included in the summand. Difference in the distribution of ${\bar{\varphi }}_{j}$ between well-fed and starved animals was tested for statistical significance.

### Statistics

Statistical analyses were performed in R (version 3.6.0, The R Foundation for Statistical Computing) on the platform x86_64-apple-darwin15.6.0 (64-bit). Differences in calcium signals of neurons, cross-correlation function and Fourier amplitude between well-fed and starved animals were tested for statistical significance by using Brunner–Munzel test that does not require the assumption of the normality and homoscedasticity of the distributions. To avoid Type I errors caused by multiple tests for a dataset, we adjusted α levels based on Bonferroni correction (see the legends of figures for exact α levels). For the statistical analyses of phase lag between AFD and AIY, we used statistical tests applicable for circular data. To test whether the distribution of phase lag is biased to a certain value, we used *V* test (modified Rayleigh test) for uniformity, where the alternative hypothesis is a unimodal distribution with a specified mean direction and unknown mean resultant length. *V* test requires von Mises distribution of the data, so we checked the goodness of fits for the von Mises distribution by using Watson’s test before performing *V* test. To test whether the data of phase lag of well-fed and starved animals are generated from the same population, we performed a non-parametric test, Mardia–Watson–Wheeler test, for homogeneity of the data. In Mardia–Watson–Wheeler test, the difference between the samples was detected in either the mean or the variance of the data. The list of data structure, type of test, and confidence intervals in the all analyses were shown in the statistical table (Table 1).

**Table 1:** Statistical table

*p* value ID	Data structure	Type of test	Confidence interval
a	Unknown	Brunner–Munzel test	0.3275941, 0.7290465
b	Unknown	Brunner–Munzel test	0.05490425, 0.36501762
c	Unknown	Brunner–Munzel test	0.2356553, 0.6452041
d	Unknown	Brunner–Munzel test	0.1743477, 0.5463554
e	Unknown	Brunner–Munzel test	0.6141842, 0.9346439
f	Unknown	Brunner–Munzel test	0.3564806, 0.7489882
g	von Mises	*V* test (modified Rayleigh test)	0.09356416, 0.48786224
h	von Mises	*V* test (modified Rayleigh test)	–2.86433755, –2.45365845
i	Unknown	Mardia–Watson–Wheeler test	
j	von Mises	*V* test (modified Rayleigh test)	2.47406820, 2.66296980
k	von Mises	*V* test (modified Rayleigh test)	2.79623445, 2.94432155
l	Unknown	Mardia–Watson–Wheeler test	
m	Unknown	Brunner–Munzel test	0.1165913, 0.6959087
n	Unknown	Brunner–Munzel test	–0.0812276, 0.4015401
o	Unknown	Brunner–Munzel test	0.1240502, 0.7118873
p	Unknown	Brunner–Munzel test	0.05295943, 0.67360307
q	Unknown	Brunner–Munzel test	–0.1027711, 0.2199586
r	Unknown	Brunner–Munzel test	0.04407648, 0.57311102
s	Unknown	Mardia–Watson–Wheeler test	
s1	von Mises	*V* test (modified Rayleigh test)	–0.10910136, 0.33118656
s2	von Mises	*V* test (modified Rayleigh test)	No value because of no bias in the distribution
t	Unknown	Mardia–Watson–Wheeler test	
u	Unknown	Brunner–Munzel test	0.01713815, 0.51619519
v	Unknown	Brunner–Munzel test	–0.04430851, 0.12635979
w	Unknown	Brunner–Munzel test	0.05222952, 0.62469356
x	Unknown	Brunner–Munzel test	–0.05517292, 0.22953190
y	Unknown	Mardia–Watson–Wheeler test	
z	Unknown	Brunner–Munzel test	0.3431445, 0.6264604
aa	Unknown	Brunner–Munzel test	0.0953605, 0.3149738
bb	Unknown	Brunner–Munzel test	0.3236050, 0.6049664
cc	Unknown	Brunner–Munzel test	0.2536403, 0.5330661
dd	Unknown	Brunner–Munzel test	0.1673401, 0.4304309
ee	Unknown	Brunner–Munzel test	0.2763699, 0.5539873

The list of data structure, type of test, and confidence interval in all analyses are shown; *p* value ID represents superscript lower-case letter of *p* value provided in the result section and the figures.

### Code accessibility

The code/software described in the paper is freely available online at https://github.com/ikedamuneki/ThermotaxisAnalysis.

## Results

### Response timing of AIY interneurons to the activities of AFD thermosensory neurons is altered by past feeding experiences

To investigate AFD-AIY response interaction and its alteration by feeding experience, we performed simultaneous calcium imaging of AFD and AIY under temporal thermal stimuli for well-fed and starved animals. Calcium indicators R-CaMP2 (Inoue et al., 2015) and GCaMP3 (Tian et al., 2009) were expressed in AFD and AIY, respectively. Well-fed and starved animals were conditioned at 20°C for 3 h with and without food, respectively, immobilized on a temperature controllable stage, and subjected to several types of thermal stimuli (Fig. 1*A*).

We monitored AFD and AIY calcium responses when delivering oscillatory thermal ramp ranging from 17.5°C to 21.0°C (designated as *Osci1721*). AFD calcium signals of well-fed animals were increased with warming and decreased with cooling, indicating that they were synchronized with thermal oscillation (Fig. 1*B*). AIY of well-fed animals appeared to be positively correlated with AFD at lower temperature (17.5–18.0°C; Fig. 1*B*), whereas AIY appeared to exhibit time-lagged responses to AFD at higher temperature (18.0–21.0°C; Fig. 1*B*). To quantify the temporal correlation between AFD and AIY with particularly focusing on time lags, we calculated cross-correlation function that represents the similarity of two signals as a function of time and time lag *τ*. In well-fed animals, the cross-correlation function showed higher values at *τ* = 0 at lower temperature (17.5–18.0°C; Fig. 1*D*), whereas, at higher temperature (18.0–21.0°C), the cross-correlation function showed lower values at *τ* = 0 and higher values at *τ* = 15 (Fig. 1*D*). This indicates that the signals of AFD and AIY of well-fed animals simultaneously took high values at lower temperature (17.5–18.0°C), whereas AIY was activated ∼15 s after AFD activation at higher temperature (18.0–21.0°C).

Starved animals exhibited AFD-AIY interaction distinct from well-fed animals especially at lower temperature. AFD of starved animals showed similar activity pattern to that of well-fed animals (Fig. 1, compare *C* and *B*), indicating that starvation did not alter the AFD response properties over the entire range of temperature we tested. In agreement with the previous reports (Kodama et al., 2006; Tsukada et al., 2016), statistical test for the peak values of AFD signals suggested that AFD activity in response to both lower (17.5–18.0°C) and higher temperature (18.0–21.0°C) was not affected by starvation (Fig. 1*F*, *p*^a^, *p*^c^). AIY of starved animals responded ∼15 s after AFD in the entire temperature range we tested (17.5–21.0°C; Fig. 1*C*). The statistical test for the values of AIY signals when AFD signals took peaks revealed that AIY responses of starved animals significantly differed from those of well-fed animals at lower temperature (17.5 − 18.0°C; Fig. 1*F*, *p*^b^), whereas no significant difference was observed at higher temperature (18.0–21.0°C; Fig. 1*F*, *p*^d^) . The cross-correlation function of starved animals showed lower values at around *τ* = 0 and higher values at *τ* = 15 over entire temperature range we tested (17.5–21.0°C; Fig. 1*E*). The statistical test for the cross-correlation function at *τ* = 15 showed that AFD-AIY interaction of starved animals significantly differed from that of well-fed animals at lower temperature (17.5–18.0°C; Fig. 1*G*, *p*^e^). By contrast, we did not find significant difference at higher temperature (18.0–21.0°C; Fig. 1*G*, *p*^f^). The analyses of calcium signals and cross-correlation function suggest that starvation altered AFD-AIY temporal interaction at lower temperature (17.5–18.0°C), but not at higher temperature (18.0–21.0°C).

To quantify the time lags between AFD and AIY signals in more detail, we calculated the phase shift between the derivatives of AFD and AIY activities by Hilbert transform. Hilbert transform enables us to calculate the instantaneous phase shift *φ*(*t*), where −π≤φ(t)≤π. *φ* is equal to 0 when the derivatives of AFD and AIY show in-phase synchronization, and *φ* is equal to ± *π* when the derivatives of AFD and AIY show anti-phase synchronization*. φ* is positive when AIY is activated later than AFD response, and *φ* is negative when AIY activity is followed by AFD response. The phase shift between the derivatives of AFD and AIY signals in well-fed animals at lower temperature (17.5–18.0°C) was distributed with bias toward 0 (*p*^g^ = 3 × 10^−4^ by *V* test; Fig. 1*H*). On the other hand, the phase shift of starved animals tended to be distributed with bias toward *π* (*p*^h^ = 1.3 × 10^−3^ by *V* test; Fig. 1*H*). The statistical test revealed a significant difference in the distribution of phase shift between well-fed and starved animals (Fig. 1*H*, *p*^i^). These results suggest that AFD and AIY of well-fed animals exhibit in-phase synchronization at lower temperature (17.5–18.0°C), whereas starved animals exhibit anti-phase synchronization. By contrast, both well-fed and starved animals showed anti-phase synchronization at higher temperature (18.0–21.0°C; *p*^j^ = 3.11 × 10^−6^ and *p*^k^ = 2.97 × 10^−9^ by *V* test; Fig. 1*I*), and there was no significant difference (Fig. 1*I*, *p*^l^). These findings suggest that starvation delays the responses of AIY to AFD at lower temperature (17.5–18.0°C) by π.

**Figure 2.**
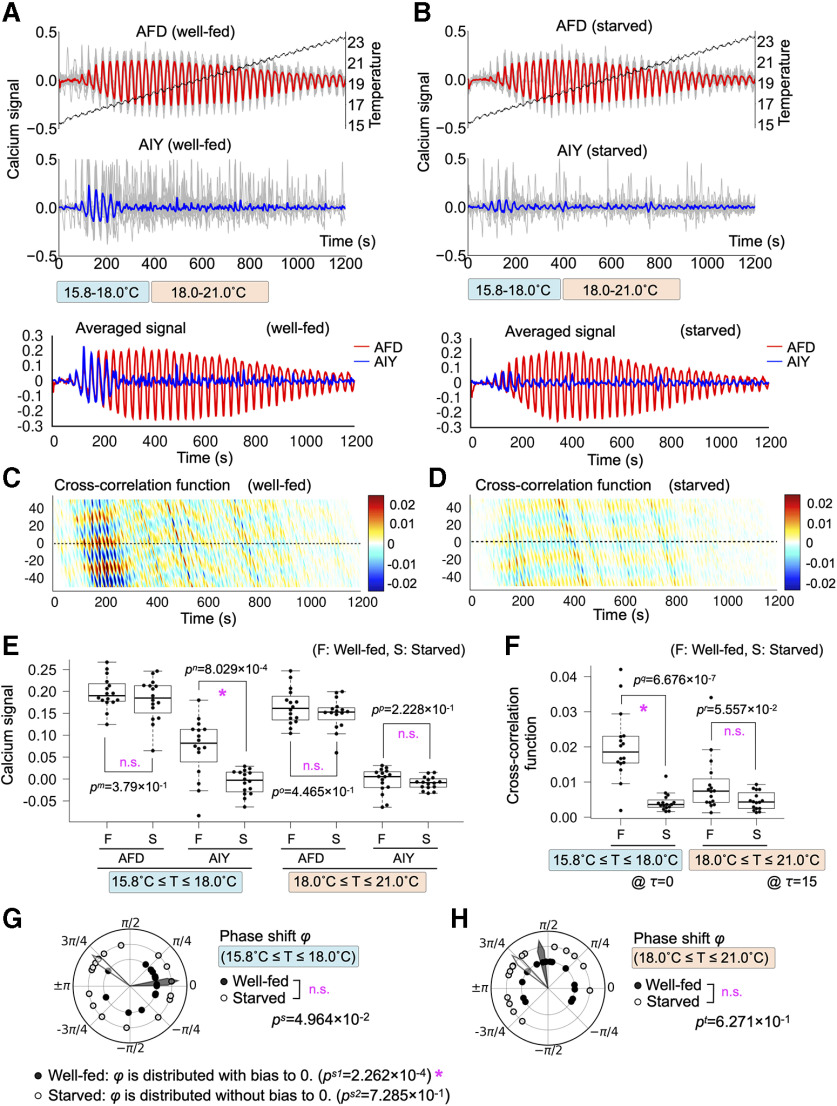
AFD-AIY temporal interaction in response to oscillatory thermal ramp from 15.8°C to 23.0°C (*Osci1523*) was altered by starvation. ***A***, AFD-AIY calcium responses of well-fed animals under thermal stimuli *Osci1523* (*n* = 16). Dashed black line indicates mean of the thermal stimuli. The gray lines indicate individual traces of calcium signals of AFD and AIY. The red and blue lines represent the mean of individual AFD and AIY signals, respectively. The blue and red bars indicate the temperature range of lower (15.8–18.0°C) and higher one (18.0–21.0°C). ***B***, AFD-AIY calcium responses of starved animals under thermal stimuli *Osci1523* (*n* = 16). ***C***, Cross-correlation function between AFD and AIY activities for well-fed animals. ***D***, Cross-correlation function between AFD and AIY activities for starved animals. ***E***, Differences in AFD and AIY calcium signals between well-fed and starved animals. Four datasets on the left: the results of AFD and AIY signals at lower temperature (15.8–18.0°C). The peak values of AFD signals and the values of AIY signals when AFD signals reach their peaks. Four datasets on the right: the results of AFD and AIY signals at higher temperature (18.0–21.0°C). Differences in AFD and AIY signals between well-fed and starved animals were tested for statistical significance by using Brunner–Munzel test; *p* values <0.05/5 = 0.01 (1%) were considered statistically significant. Asterisk indicates *p *<* *0.01 (statistically significant), and n.s. indicates *p *≥* *0.01 (not significant). ***F***, Difference in the cross-correlation function between well-fed and starved animals. Brunner–Munzel test was used for statistical analysis; *p* values <0.05/5 = 0.01 (1%) were considered statistically significant. Asterisk indicates *p *<* *0.01 (statistically significant), and n.s. indicates *p *≥* *0.01 (not significant). ***G***, Difference in phase shift between AFD and AIY responses at lower temperature (15.8–18.0°C). The black dots indicate phase shift between AFD and AIY responses for well-fed animals, and white dots are for starved animals. The black and white arrowheads represent mean directions of individual phase shift vectors for well-fed and starved animals, respectively. Mardia–Watson–Wheeler test was used for statistical analysis; *p* values <0.05/5 = 0.01 (1%) were considered statistically significant. Asterisk indicates *p *<* *0.01 (statistically significant), and n.s. indicates *p *≥* *0.01 (not significant). ***H***, Difference in phase shift between AFD and AIY at higher temperature (18.0–21.0°C). Quantifications and statistical tests were done in the same manner as ***G***.

To investigate whether AFD-AIY interaction observed above is preserved under other thermal stimuli, we delivered another type of oscillatory thermal ramp ranging from 15.0°C to 23.0°C (designated as *Osci1523*), which covers wider range of temperature than *Osci1721*. AFD of well-fed animals increased their calcium signals around 16°C, and the calcium dynamics was synchronous with the thermal stimuli (Fig. 2*A*). AIY of well-fed animals was tightly coupled with AFD at lower temperature (16–18°C; Fig. 2*A*). Cross-correlation function calculated for well-fed animals showed higher values at τ = 0 at lower temperature (16–18°C; Fig. 2*C*), suggesting that AFD and AIY signals simultaneously take high values at lower temperature (16.0–18.0°C).

Starved animals exhibited distinct AFD-AIY interaction. AFD of starved animals exhibited similar activity pattern to that of well-fed animals (Fig. 2*B*). The statistical test for the peak values of AFD calcium signals revealed that there was no significant difference in AFD responses between well-fed and starved animals (Fig. 2*E*, *p*^m^). Although AIY of starved animals were coupled with AFD at lower temperature (16–18°C), AIY responses appeared to be delayed relative to AFD responses (Fig. 2*B*), as compared with those of well-fed animals. Indeed, in starved animals, the values of AIY signals at AFD peaks significantly differed from those of well-fed animals (Fig. 2*E*, *p*^n^), suggesting that AIY responses to AFD at lower temperature (16–18°C) is altered by starvation. Cross-correlation function of starved animals showed higher values at *τ* ≠ 0, and the time lag τ, where the function took high values, changed transiently at lower temperature (16–18°C; Fig. 2*D*), suggesting that AIY responses are gradually delayed in relation to AFD responses. The statistical test for the values of cross-correlation function at *τ* = 0 at lower temperature (16–18°C) found significant difference between well-fed and starved animals (Fig. 2*F*, *p*^q^). These results suggest that AFD-AIY temporal interaction at lower temperature (16–18°C) is changed by starvation. On the other hand, AFD-AIY interaction was not affected by starvation at higher temperature (18–21°C; Fig. 2*E*, *p*^o^, *p*^p^; *F*, *p*^r^). Given these observations, the starvation-induced neuronal alteration observed under *Osci1721* is preserved under *Osci1523*, suggesting that AFD-AIY alteration at lower temperature does not depend on the history of thermal stimuli.

To further quantify the AIY response timing, phase shift between the derivatives of AFD and AIY was calculated. We could not find significant difference in the distribution of phase shift between well-fed and starved animals at lower temperature by using Mardia–Watson–Wheeler test (*p*^s^ = 4.96 × 10^−2^; Fig. 2*G*). Another statistical test for anisotropy of the phase shift distribution however revealed that AFD-AIY response of well-fed and starved animals tended to be different. The phase shifts in well-fed animals was significantly distributed with bias to 0 (*p*^s1^ = 2.262 × 10^−4^ by *V* test), whereas we could not detect the significant bias in starved animals (*p*^s2^ = 7.285 × 10^−1^ by *V* test). Similar to the results with the stimuli *Osci1721*, starvation altered the timing of AIY responses to AFD responses at lower temperature (16–18°C), although starvation did not modify the AFD-AIY temporal interaction at higher temperature (18–21°C; Fig. 2*H*, *p*^t^). The result at lower temperature under the stimulus *Osci1523* seems to be slightly different from the result under *Osci1721*, possibly due to the effect of whether the animals experienced thermal stimulation around 15°C that is much lower than their cultivation temperature (20°C).

The above two types of thermal stimuli (*Osci1721* and *Osci1523*) contain long-term trend of linear warming. To examine whether starvation-induced AFD-AIY alteration observed above is also exhibited without linear warming, we delivered thermal oscillation around 16.5–17°C (*Osci17*) without linear warming. The thermal oscillation was repeated 15 times per animal with 0.025 Hz. To assess stimuli-related neuronal activities, the calcium signals were segmented into several time windows, which were time-locked to the peaks of thermal stimuli. In addition, the time axis of each time window was redefined such that thermal oscillation peaks at time 0.

**Figure 3.**
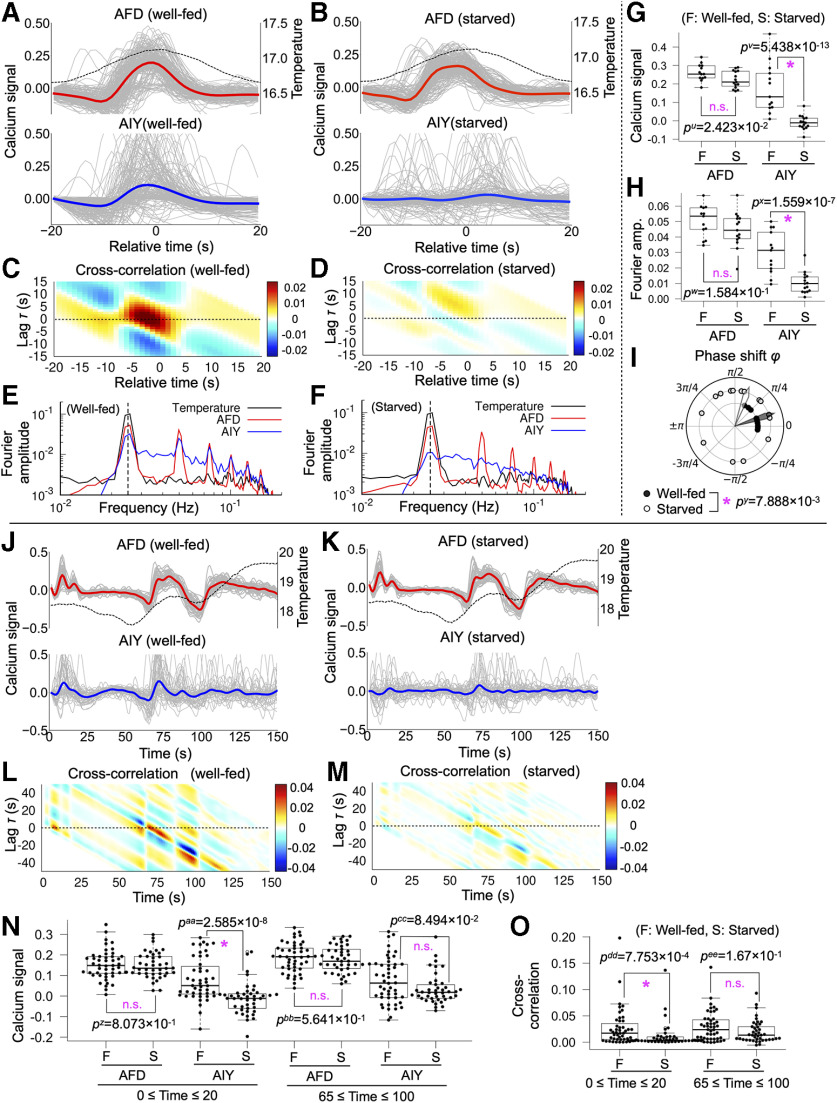
AFD-AIY temporal interaction in response to thermal oscillation around 17°C (*Osci17*) and naturally fluctuated thermal stimuli (*Fluc1719*) was altered by starvation. ***A***, AFD-AIY calcium responses of well-fed animals under thermal stimuli *Osci17* (*n* = 13). Dashed black line indicates mean of the thermal stimuli. The gray lines indicate individual traces of calcium signals of AFD and AIY. The red and blue lines represent the mean of individual AFD and AIY signals, respectively. ***B***, AFD-AIY calcium responses of starved animals under thermal stimuli *Osci17* (*n* = 15). ***C***, Cross-correlation function between AFD and AIY activities for well-fed animals. ***D***, Cross-correlation function between AFD and AIY activities for starved animals. ***E***, Fourier amplitude spectrum of temperature (black), AFD (red), and AIY activities (blue) observed in well-fed animals. Vertical dashed line represents frequency of thermal oscillation (0.025 Hz). ***F***, Fourier amplitude spectrum of temperature (black), AFD (red), and AIY activities (blue) observed in starved animals. ***G***, Differences in AFD and AIY calcium signals between well-fed and starved animals. Two datasets on the left: the results of AFD signals. Two datasets on the right: the results of AIY signals. Differences in AFD and AIY signals between well-fed and starved animals were tested for statistical significance by using Brunner–Munzel test; *p* values <0.05/3 = 0.0167 (1.67%) were considered statistically significant. Asterisk indicates *p *<* *0.0167 (statistically significant), and n.s. indicates *p *≥* *0.0167 (not significant). ***H***, Differences in Fourier amplitude of AFD and AIY activities between well-fed and starved animals. Brunner–Munzel test was used for statistical analysis; *p* values <0.05/3 = 0.0167 (1.67%) were considered statistically significant. Asterisk indicates *p *<* *0.0167 (statistically significant), and n.s. indicates *p *≥* *0.0167 (not significant). ***I***, Difference in phase shift between AFD and AIY responses. The black dots indicate phase shift between AFD and AIY responses for well-fed animals, and white dots are for starved animals. The black and white arrowheads represent mean directions of individual phase shift vectors for well-fed and starved animals, respectively. Mardia–Watson–Wheeler test was used for statistical analysis; *p* values <0.05/3 = 0.0167 (1.67%) were considered statistically significant. Asterisk indicates *p *<* *0.0167 (statistically significant), and n.s. indicates *p *≥* *0.0167 (not significant). ***J***, AFD-AIY calcium responses of well-fed animals under thermal stimuli *Fluc1719* (*n* = 48). Dashed black line indicates mean of the thermal stimuli. The gray lines indicate individual traces of calcium signals of AFD and AIY. The red and blue lines represent the mean of individual AFD and AIY signals, respectively. ***K***, AFD-AIY calcium responses of starved animals under thermal stimuli *Fluc1719* (*n* = 42). ***L***, Cross-correlation function between AFD and AIY activities for well-fed animals. ***M***, Cross-correlation function between AFD and AIY activities for starved animals. ***N***, Differences in AFD and AIY calcium signals between well-fed and starved animals. Four datasets on the left: the results of AFD and AIY signals at 0 ≤ time ≤ 20 (18°C). The peak values of AFD signals and the values of AIY signals when AFD signals reach their peaks. Four datasets on the right: the results of AFD and AIY signals at 65 ≤ time ≤ 100 (18–18.5°C). Differences in AFD and AIY signals between well-fed and starved animals were tested for statistical significance by using Brunner–Munzel test; *p* values <0.05/2 = 0.025 (2.5%) were considered statistically significant. Asterisk indicates *p *<* *0.025 (statistically significant), and n.s. indicates *p *≥* *0.025 (not significant). ***O***, Difference in the cross-correlation function between well-fed and starved animals. Four datasets on the right, Cross-correlation at 0 ≤ time ≤ 20 (18°C). Four datasets on the right, Cross-correlation at 65 ≤ time ≤ 100 (18°C). Brunner–Munzel test was used for statistical analysis; *p* values <0.05/2 = 0.025 (2.5%) were considered statistically significant. Asterisk indicates *p *<* *0.025 (statistically significant), and n.s. indicates *p *≥* *0.025 (not significant).

AIY of well-fed animals increased and decreased their calcium signals in accordance with AFD activities (Fig. 3*A*). Consistent with this observation, cross-correlation function of well-fed animals showed higher values at approximately *τ* = 0 (Fig. 3*C*), suggesting that AIY responses are positively correlated with AFD responses without time lag. Fourier analysis showed that both AFD and AIY signals of well-fed animals contain strong vibration components at the frequency of the thermal oscillation ω* = 0.025 Hz (Fig. 3*E*). On the other hand, AIY of starved animals showed non-synchrony activities (Fig. 3*B*). Cross-correlation function of starved animals did not show high value at *τ* = 0 (Fig. 3*D*). The statistical test for the AFD and AIY signals showed that AFD response property is not affected by starvation, whereas AIY responses to AFD responses are altered by starvation (Fig. 3*G*, *p*^u^*, p*^v^). The vibration component of AIY at ω* was significantly changed by starvation (Fig. 3*F*,*H*, *p*^x^), but not for AFD (Fig. 3*H*, *p*^w^). We also detected a significant difference in the phase shift between well-fed and starved animals (Fig. 3*I*, *p*^y^), and AIY responses of starved animals appeared to be delayed relative to AFD responses. These results suggest that starvation alters AFD-AIY interaction, and tends to delay AIY responses to AFD at around 17°C.

To examine the neuronal activities under a naturally fluctuated thermal stimulus, we delivered a thermal stimulus generated by Gaussian white noise (17.5–19.5°C). AFD of well-fed animals showed calcium responses on temperature rises at 10 ≤ time ≤ 20 (18.0°C), 65 ≤ time ≤ 100 (18.0–18.5°C), and time ≥ 100 (18.0–19.5°C) (Fig. 3*J*). AIY of well-fed animals increased their calcium signals around 10 ≤ time ≤ 20 (18.0°C) and 65 ≤ time ≤ 100 (18.0–18.5°C) in response to AFD (Fig. 3*J*). The cross-correlation function at *τ* = 0 was remarkably increased at these two regions (Fig. 3*L*). Although the response property of AFD was not altered by starvation (Fig. 3*K*,*N*, *p*^z^*, p*^bb^), AIY responses at 10 ≤ time ≤ 20 (18.0°C) was significantly altered by starvation (Fig. 3*J*,*K*,*N*, *p*^aa^), and tends to be opposite to AFD response. The cross-correlation function at 10 ≤ time ≤ 20 (18.0°C) was also altered by starvation, significantly (Fig. 3*L*,*M*,*O*, *p*^dd^). These results suggest that starvation affects AFD-AIY interaction at lower temperature (18.0°C) even under naturally fluctuated thermal stimulus. Consistent with the results obtained under other types of thermal stimuli, we did not find significant alteration at higher temperature (18.0–18.5°C; Fig. 3*N*, *p*^cc^, *O*, *p*^ee^).

By conducting AFD-AIY simultaneous calcium imaging under different types of thermal stimuli, we have shown that past feeding experiences altered temporal interaction between AFD thermosensory neurons and AIY interneurons. For 20°C-cultivated animals, the starvation delayed or anti-synchronized AIY responses to AFD responses at lower temperature (16.0–18.0°C), whereas AFD-AIY interaction was not affected by starvation at higher temperature (18.0–21.0°C).

### Starvation altered curving behavior on a linear thermal gradient

**Figure 4.**
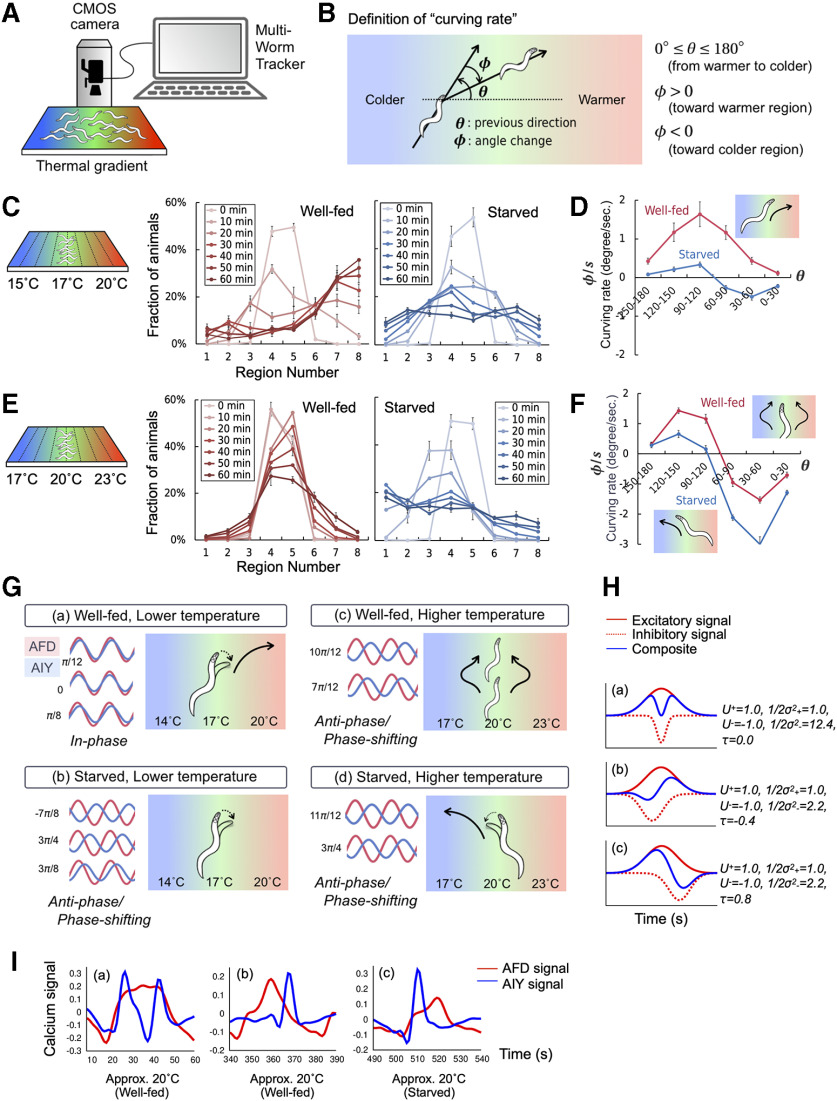
Starvation-induced modulation of curving behavior on a thermal gradient and its correlation with AFD-AIY activities. ***A***, The schematic figures of MWT. ***B***, Definition of curving behavior on a thermal gradient. ***C***, Thermotaxis behavior of 20°C-conditioned animals on 17°C-centered thermal gradient. The left panel with red lines indicates the distribution of well-fed animals in each section of the thermotaxis assay plate (*n* = 9). The right panel with blue lines is for starved animals (*n* = 5). The values indicate means ± SEM, and as the color of line changes from light to dark, it indicates that time passed since the starts of assays. ***D***, Curving rate of well-fed and starved animals measured from 10 to 20 min after placing the animals on the assay plate. ***E***, Thermotaxis behavior of 20°C-conditioned animals on 20°C-centered thermal gradient (well-fed *n* = 6, starved *n* = 6). ***F***, Curving rate of well-fed and starved animals measured from 0 to 10 min after placing the animals on the assay plate. ***G***, Relationship between temporal AFD-AIY interaction and behavioral outputs on a thermal gradient. The schematic diagram of neuronal activity patterns is displayed next to the diagrams showing the relevant animal locomotion on a thermal gradient. The red and blue traces represent AFD and AIY activities, respectively. The numerical values written next to the diagram of AFD-AIY activities represents the mean direction of phase shit. The three activity patterns observed under the thermal stimulus *Osci1721*, *Osci152*3, and *Osci17* are shown in order from the top (***a***, ***b***). The two activity patterns observed under the thermal stimulus *Osci1721* and *Osci1523* are shown in order from the top (***c***, ***d***). ***H***, Various patterns of interneuron activities can be generated by composing excitatory and inhibitory signals from sensory neurons. The red and blue lines represent excitatory and inhibitory signals written by Gaussian function, respectively. The numerical values indicate the parameters of Gaussian function. ***I***, Neuronal activity patterns similar to the numerically calculated patterns were observed in the experiments.

To investigate the behavioral outputs relevant to AFD-AIY temporal interaction, we performed quantitative behavioral analysis for freely moving animals on a linear thermal gradient. *C. elegans* is known to build their behavioral strategies by combining several behavioral components (Croll, 1975; Pierce-Shimomura et al., 1999; Iino and Yoshida, 2009; Kim et al., 2011; Schild and Glauser, 2013; Salvador et al., 2014). By using MWT system (Swierczek et al., 2011; Ikeda et al., 2018; Nakano et al., 2020), we analyzed frequencies of the behavioral components in thermotaxis (Fig. 4*A*).

To seek the behavioral components that are affected by starvation at lower temperature (16.0–18.0°C), we analyzed the behaviors of 20°C-cultivated animals on 17°C-centered thermal gradient (Fig. 4*C*). Well-fed animals migrated toward their cultivation temperature, whereas starved animals lost the attraction toward the cultivation temperature and dispersed on the thermal gradient (Fig. 4*C*). Among several behavioral components, we focused on curving behavior (Fig. 4*B*), because curving behavior, which is a directional changing during forward running, is clearly different between well-fed and starved animals and is thought to be regulated by AIY interneurons (discussed later). Well-fed animals showed biased curving toward warmer region when both crawling up and down the thermal gradient (Fig. 4*D*). On the other hand, starved animals did not show curving behavior that drives migration toward warmer region, but rather showed weak curving behavior to remain at around initial positions (Fig. 4*D*). Taken together with the results of AFD-AIY calcium imaging described above, in-phase AFD-AIY interaction observed for well-fed animals at lower temperature correlates with the curving behavior toward warmer region. On the other hand, anti-phase or phase-shifted AFD-AIY interaction observed for starved animals at lower temperature correlates with loss of attraction to the cultivation temperature.

To seek the behavioral components affected by starvation at higher temperature (18.0–21.0°C), we analyzed the behaviors of 20°C-cultivated animals on 20°C-centered thermal gradient (Fig. 4*E*). Well-fed animals remained at around cultivation temperature, whereas starved animals first migrated toward colder region and then dispersed on the thermal gradient (Fig. 4*E*). Although starvation did not alter the AFD-AIY interaction at higher temperature (18–21°C), we found that starvation affected the curving behavior at that temperature.

## Discussion

In the present study, we investigated the relationship between AFD-AIY interaction and starvation-induced preference alteration in thermotaxis. Based on simultaneous AFD-AIY calcium imaging and quantitative behavioral analysis, we showed that starvation alters temporal interaction between AFD and AIY, leading to switch the behavioral preference in starved animals. Our results suggested that the time lag or phase shift of AFD-AIY communication encodes thermal preference.

We found that past feeding experience altered temporal interaction between AFD thermosensory neurons and AIY interneurons at lower temperature. This alteration of neuronal communication is likely to be critical for behavioral regulation, since previous reports showed that the activity of AIY is tightly coupled with *C. elegans* locomotion (Kocabas et al., 2012; Li et al., 2014). The calcium signals of AIY interneurons encode animals’ speed of forward running (Li et al., 2014), and optogenetic manipulation of AIY activity showed that AIY excitation or inhibition forces animals to run toward biased direction (Kocabas et al., 2012). This biased running was observed depending on the timing of AIY activities and animals’ head swinging (Kocabas et al., 2012).

Our present findings, together with these previous knowledges, propose how well-fed and starved animals regulate appetitive and aversive behavior through AFD-AIY temporal interaction. When an animal is placed at lower temperature (17°C) on a thermal gradient, the animal senses temperature rises and falls in accordance with its head swinging whereby AFD calcium signals increase and decrease, respectively. This sensory processing is not altered by past feeding experience, because AFD response property was not affected by starvation (Figs. 1-3).

However, AIY activities in response to AFD was modified by starvation experience. Subsequently to the responses of AFD, AIY of well-fed animals show in-phase synchronization with AFD. When AIY calcium signals increase at the same time as an animal swings its head toward warmer region, then biased forward running is promoted toward warmer region (Fig. 4*Ga*). Conversely, the forward running is suppressed toward colder region, because AIY calcium signals decrease when an animal swings its head toward colder region (Fig. 4*Ga*). This interpretation is in agreement with the previous studies on the relationship between AFD-AIY communication and thermotaxis (Luo et al., 2014; Hawk et al., 2018; Nakano et al., 2020). On the other hand, for starved animals, AIY responses are delayed to AFD responses or are synchronized in anti-phase with the AFD responses (Figs. 1-3), thereby suppressing migration toward warmer region (Fig. 4*Gb*).

Our results also showed that past feeding experience did not modify AFD-AIY interaction at higher temperature (Figs. 1-3). However, we found that the curving behavior at higher temperature that can be driven by AIY was affected by past feeding experiences (Fig. 4*F*). To understand the inconsistency between neural activity and behavior at higher temperature, we emphasize the qualitative difference in the behavioral strategy between starved and well-fed animals. The starved animals migrated toward colder region (navigation), whereas well-fed animals remained themselves at their cultivation temperature (isothermal tracking). The navigation and isothermal tracking might employ different types of neural computation. Navigation strategy can be achieved by a framework in which AFD senses the temperature according to worm’s head swing, and AIY biases the worm’s direction of the movement in response to AFD. Indeed, AFD-AIY anti-phase-like activities observed in starved animals at higher temperature can explain the navigation toward colder region, since AIY calcium signals were increased with cooling and decreased with warming (Fig. 4*Gd*). However, isothermal tracking might require highly-regulated sensorimotor coupling by crosstalk between upstream and downstream neurons. In particular, worms need to integrate their head swing, position and temporal thermal stimuli in a certain time window and fine-tune their behavior to remain at around the cultivation temperature. *C. elegans* is thought to execute sensorimotor integration during crawling along isotherms on a thermal gradient, and such a sensorimotor control can be computed by RIA interneurons (Hendricks et al., 2012; Liu et al., 2018; Ouellette et al., 2018), a postsynaptic partner of AIY (White et al., 1986). We could not explain the behavior solely by AFD-AIY activities (Fig. 4*Gc*), probably due to a lack of insights to the activity patterns of further downstream neurons in the thermotaxis circuit (Mori and Ohshima, 1995; Ikeda et al., 2018). Simultaneous calcium imaging for AFD, AIY, and RIA and other neurons will uncover a neural logic for the isothermal tracking that allows animals to remain at around cultivation temperature.

Previous literatures on AFD-AIY interaction in response to thermal stimuli have shown that AIY interneurons regulate thermotaxis behavior by altering their response amplitude or frequency depending on stimulus contexts (Kuhara et al., 2011; Hawk et al., 2018; Goodman and Sengupta, 2019). In addition to that aspect, we provided here a new mechanism by which temperature preference can be encoded by the phase shift between AFD and AIY activities. From the above results, AIY neurons appear to communicate with AFD neurons by various coding protocols including amplitude, frequency and phase coding to process sensory information and execute behavioral response. AFD thermosensory neurons are known to interact with AIY via both excitatory and inhibitory signals (Kuhara et al., 2011). Hence, such various coding styles can be achieved by arithmetic operations of excitatory and inhibitory signals. For example, AIY signals can be formed by composite waveforms of excitatory and inhibitory signals that are transmitted from AFD. Indeed, superposition of excitatory and inhibitory signals, for example, Gaussian function of time written as $U^{+}\text{exp}\;(-t^{\text{2}}/\text{2}\sigma_{+}^{2})+U^{-}\text{exp}(-{\left(t-\tau \right)}^{\text{2}}/\text{2}\sigma_{-}^{2})$, can easily generate various activity patterns (Fig. 4*Ha*, 1:2 resonance, *Hb*, post-peak responding, *Hc*, pre-peak responding). AIY activity patterns are formed depending on time lag (*τ*) of inhibitory signals relative to excitatory signals, sharpness of signals (*σ*_+_, *σ*_-_), and signal magnitude (*U*^+^ > 0, *U*^–^ < 0). These activity patterns generated above were actually observed in AFD-AIY imaging experiments under several conditions (Fig. 4*Ia–c*). Recently, it has been reported that there is a complex relationship between neurotransmitter inputs and calcium responses in AIY (Ashida et al., 2019). Given these observations, it is plausible that timing, sharpness and magnitude of synaptic inputs shape multiple activity patterns in postsynaptic neurons, and might regulate various behavioral outputs. AFD is known to communicate with AIY via both neuropeptide and glutamate (Narayan et al., 2011; Ohnishi et al., 2011). The peptidergic synaptic transmission is excitatory signal (Narayan et al., 2011), on the other hand, the glutamatergic synaptic transmission is inhibitory signal (Ohnishi et al., 2011). The previous study showed that UNC-31, *C. elegans* ortholog of calcium-dependent activator protein required for the secretion of neuropeptide (Speese et al., 2007) and EAT-4, *C. elegans* homolog of sodium-dependent inorganic phosphate cotransporter required for glutamatergic transmission (Lee et al., 1999) function in AFD to regulate the excitatory-inhibitory balance onto AIY (Nakano et al., 2020). The opposing signals from AFD via UNC-31 and EAT-4 might generate various patterns of postsynaptic response in AIY as we theoretically predicted above. Regulating the threshold for neurotransmitter release and the rate of recovery or degradation might control the timing and sharpness of these opposing signals.

Theoretical approaches have suggested that balance or relative temporal relation between excitation and inhibition participates in signal propagation through neural network (Vogels and Abbott, 2009; Kremkow et al., 2010; Shinozaki et al., 2010). Indeed, correlation between excitatory and inhibitory signals has been observed in the rat somatosensory cortex during spontaneous activities and evoked activities by whiskers’ stimulation (Okun and Lampl, 2008). Moreover, temporal shift of excitation relative to inhibition in rat somatosensory cortex is known to be crucial for direction selectivity (Wilent and Contreras, 2005). An optogenetic evidence of mouse hippocampal neurons has also suggested that neural information processing is controlled by excitation-inhibition balance and delay (Bhatia et al., 2019). Furthermore, the leech nervous system recruits excitatory-inhibitory balance to adjust the gain of the neural circuit underlying sensory response behavior (Baca et al., 2008), implicating that excitatory-inhibitory interaction takes important role in neural information processing not only in vertebrates but also invertebrates. We assume that the neural activities with phase shifts or time lags enable the nervous system to process sensory information with proper latency, thereby allowing animals to respond to ambient stimuli flexibly. Our investigation into one of the most tractable nervous system should shed light on the neural mechanism by which temporal interaction between sensory neurons and interneurons encode animals’ sensory preferences, and regulate attraction and avoidance behaviors.

Emotional processing of pleasure and unpleasure is crucial for animals to motivate their behaviors. Animals are able to assign positive or negative hedonic value to a stimulus based on their past experiences, thereby surviving in ever-changing environments. We showed here that past feeding experiences altered interaction between thermosensory neurons (AFD) and interneurons (AIY), thereby switching their behavioral preference in regard to temperature. The numerous studies have proposed several neural circuit motifs for the emotional processing (Tye, 2018): (1) a circuit with parallel information paths for positive and negative hedonic values (Root et al., 2014; Wang et al., 2018); (2) a circuit with divergent paths from a common sensory input to divergent outputs (Namburi et al., 2015; Beyeler et al., 2016, 2018); (3) a circuit wherein both positive and negative signals are transmitted from the same upstream neurons to the same downstream neurons (Barbano et al., 2016; Nieh et al., 2016; Stamatakis et al., 2016; Li et al., 2018), and so on. The neuronal mechanism we reported here belongs to the circuit motif (3). Since the response property of AFD calcium signals was not modified by past feeding experiences, the neuronal computation by the circuit (3) is likely to be done on synapses between AFD and AIY. Indeed, based on optical control of neuronal activities and genetic approaches (Kuhara et al., 2011), AFD thermosensory neurons have been known to transmit both excitatory and inhibitory signals to AIY interneurons. In addition, according to a recent literature that investigated *C. elegans* thermotaxis behavior, it has been found that the synaptic communication between AFD and AIY was opposite depending on stimulus contexts: AIY calcium signals are increased and decreased when well-fed animals sense lower and higher temperature than their cultivation temperature, respectively (Nakano et al., 2020). Moreover, the opposite communication between pairs of sensory neurons and interneurons has also been observed in another behavioral paradigm with CO_2_ modality (Guillermin et al., 2017; Rengarajan et al., 2019). Such a neuronal strategy enables animals to enrich information encoding with limited neuronal resources. Our finding should exemplify a mechanism by which small nervous systems facilitate preference switching via local neuronal computation.

## Synthesis

Reviewing Editor: Leonard Maler, University of Ottawa Decisions are customarily a result of the Reviewing Editor and the peer reviewers coming together and discussing their recommendations until a consensus is reached. When revisions are invited, a fact-based synthesis statement explaining their decision and outlining what is needed to prepare a revision will be listed below. The following reviewer(s) agreed to reveal their identity: Jonathan Pierce-Shimomura, William Schafer.

Editor comments

Both reviewers and the Editor agree that this Ms suggests a novel mechanism by which a C elegans memory of a particular temperature can be regulated whether they are starved or well fed. This is clearly an important advance in the C elegans field, but may also be interesting to a wider audience that tries to connect internal states to perception.

Statistical analysis: One reviewer notes that the authors mention a custom software package for analysis of behavioral data. The authors should clearly indicate if this is a customized version of a published “multiworm tracker” with a citation. Alternatively, if this is their own code, it should be properly validated. In any case, this likely small issue should be clarified.

Both reviews were very favourable. The second reviewer did have some detailed questions that the authors must address. I have provided edited versions of both reviews that provide clear directions as to the revisions that must be made to this Ms.

Reviewer #1

Minor issues:

Although the author’s final sentence in the abstract is likely true: “Such a neuronal strategy may enable animals to enrich information processing with limited neuronal resources.” I think they may be underselling their discovery because this third mechanism be essential for the other two in this system and in others. So it remains to be seen if this is simply a quirky mechanism for a compact nervous system or not.

It would be helpful to have a dotted black line at y=0 for the cross correlation plots to help see the shift from phase lag of zero to about 15 seconds in Fig 1D and not in Fig 1E.

Reviewer #2

The key experiments, in Figures 1-3, show that at use several different stimulus paradigms (rapid oscilliation superimposed on a gradual increase from around 18-21C, oscillation imposed on a steeper increase form 15-23C, and oscillation or random fluctuation around 17C). In all cases, at lower temperatures well-fed animals show a smaller phase shift between AFD and AIY activity than starved animals, while at higher temperatures both well-fed and starved animals show relatively large phase shifts. In Figure 1 (panels H and I) this sems very clear–at low temperatures AFD and AIY are in phase (phase shift around 0) for fed animals and antiphase (phase shift around pi) for starved animals; both are near antiphase at high temperatures. But in Figure 2, even though the authors describe the results simliarly, the actual phase shift patterns they show in panels 2G and 2H look quite similar at high and low temperatures (what looks like a phase shift of around pi/2 at both high and low temperatures, though only low temp shows statistical significance). Maybe I am missing something, but it seems like the authors should comment on why the results seem different with the two different ramp protocols.

I also found the results in Figure 4 hard to understand. At low temperatures, it makes sense that well-fed animals (where AIY activity is in phase with AFD) would show biased turning while starved animals (where AIY and AFD are out of phase) would show little bias. But I don’t understand why at high temperatures starved animals would show different behavior from fed animals when the phase shifts in Figures 1 and 2 were not significantly different (and for the gentler ramp they actually look more or less the same). Again, I don’t see anything wrong with the data but I think the authors should explain their interpretations better.

Finally, I liked the authors’ hypothesis that the phase shift differences could be the result of varying the strengths of inhibitory and excitatory transmission from AFD to AIY with different temporal dynamics. Is there any molecular evidence in support of this idea, or experiments that could test this hypothesis?

## Author Response

Here is the response letter for the manuscript entitled “Neural coding of thermal preferences in

the nematode Caenorhabditis elegans” by Hironori J. Matsuyama and Ikue Mori [Paper

#eN-NWR-0414-19].

We wish to express our sincere appreciation to Reviewing Editor and Reviewers for their insightful

comments on our manuscript. The comments have helped us significantly improve the

manuscript.

We have addressed all the comments expressed by Reviewing Editor and Reviewers, as indicated below, and hope that our explanations and revisions are satisfactory. Following are our detailed point-by-point responses to the Reviewing Editor’s and Reviewers’ comments.

Responses to Reviewing Editor

Q1. Statistical analysis: One reviewer notes that the authors mention a custom software package for analysis of behavioral data. The authors should clearly indicate if this is a customized version of a published “multiworm tracker” with a citation. Alternatively, if this is their own code, it should be properly validated. In any case, this likely small issue should be clarified.

A1. We realized that our description for the software package was inappropriate. To clarify that the software package of Multi-Worm Tracker for image capturing and processing we used in the present study is a published version (Swierczek et al., 2011) rather than a customized version, we removed the word “customized” (lines 136, 142-143 and 148). In addition, we clarified that the software package for behavioral analysis we used in the present study is a published version (Yamaguchi et al. 2018, Nakano et al. 2020, Ikeda et al., 2018), as we indicated that clearly by removing the word “customized” (line 150).

Responses to Reviewer #1

Q1. Although the author’s final sentence in the abstract is likely true: “Such a neuronal strategy may enable animals to enrich information processing with limited neuronal resources.” I think they may be underselling their discovery because this third mechanism be essential for the other two in this system and in others. So it remains to be seen if this is simply a quirky mechanism for a compact nervous system or not.

A1. We agree with the Reviewer and have modified the final sentence in the abstract to convey general importance of our results (lines 23-25): “Such a neural strategy may enable animals to enrich information processing within defined connectivity via dynamic alterations of synaptic communication.”

Q2. It would be helpful to have a dotted black line at y=0 for the cross correlation plots to help see the shift from phase lag of zero to about 15 seconds in Fig 1D and not in Fig 1E.

A2. According to Reviewer #1’s suggestion, we have added dotted black lines at y=0 for the cross-correlation plots (Fig. 1D, 1E, 2C, 2D, 3C, 3D, 3L and 3M).

Responses to Reviewer #2

Q1. The key experiments, in Figures 1-3, show that at use several different stimulus paradigms

(rapid oscilliation superimposed on a gradual increase from around 18-21C, oscillation imposed on a steeper increase form 15-23C, and oscillation or random fluctuation around 17C). In all cases, at lower temperatures well-fed animals show a smaller phase shift between AFD and AIY activity than starved animals, while at higher temperatures both well-fed and starved animals show relatively large phase shifts. In Figure 1 (panels H and I) this sems very clear–at low temperatures AFD and AIY are in phase (phase shift around 0) for fed animals and antiphase (phase shift around pi) for starved animals; both are near antiphase at high temperatures. But in Figure 2, even though the authors describe the results simliarly, the actual phase shift patterns they show in panels 2G and 2H look quite similar at high and low temperatures (what looks like a phase shift of around pi/2 at both high and low temperatures, though only low temp shows statistical significance). Maybe I am missing something, but it seems like the authors should comment on why the results seem different with the two different ramp protocols.

A1. We agree with Reviewer’s assessment with respect to the difference in AFD-AIY responses between two different thermal ramp protocols: oscillatory thermal ramp from 17˚C to 21C

(Osci1721) and one from 15˚C to 23˚C (Osci1523). We believe that the difference in the results between the two protocols are probably due to the difference in the temperature history experienced by animals. In the case of Osci1523, the animals were exposed to much lower temperature than the cultivation temperature, which may have affected the results. The corresponding description has been given in lines 360-364.

In addition, we have reanalyzed phase shift based on the time derivatives of AFD and AIY signals to measure the response timing of neurons more accurately. Accordingly, all the figures, p-values and statistical table related to the phase shift have been replaced (Fig. 1H, 1I, 2G, 2H, 3I, and Table 1). The description of the method for calculating phase shift was also modified (lines 197-200, 209,

213, 216-218, 298, 300-301, 304, 306-307 and 313). The results of the statistical analysis were the same as in the previous version, except for Fig. 2G (the data of the phase shift at lower

temperature under Osci1523, statistically tested by using Mardia-Watson-Wheeler test). However, by applying another statistical test (V-test) to the data of Fig. 2G, we found that AFD-AIY response of well-fed and starved animals tended to be different. The phase shift data of well-fed animals were significantly biased toward 0, whereas those of starved animals were not biased toward 0. Thus, we concluded that there were no changes in the results after the reanalysis. The corresponding description has been provided in lines 349-357.

Q2. I also found the results in Figure 4 hard to understand. At low temperatures, it makes sense that well-fed animals (where AIY activity is in phase with AFD) would show biased turning while starved animals (where AIY and AFD are out of phase) would show little bias. But I don’t understand why at high temperatures starved animals would show different behavior from fed animals when the phase shifts in Figures 1 and 2 were not significantly different (and for the gentler ramp they actually look more or less the same). Again, I don’t see anything wrong with the data but I think the authors should explain their interpretations better.

A2. In accordance with Reviewer #2’s comment, we have rephrased our interpretation of the discordancy between neural activity and behavior at higher temperature: “To understand the inconsistency between neural activity and behavior at higher temperature, we emphasize the qualitative difference in the behavioral strategy between starved and well-fed animals. The starved animals migrated toward colder region (navigation), whereas well-fed animals remained themselves at their cultivation temperature (isothermal tracking). The navigation and isothermal tracking might be achieved by different types of neural computation. Navigation strategy can be achieved by a framework in which AFD senses the temperature according to worm’s head swing, and AIY biases the worm’s direction of the movement in response to AFD. Indeed, AFD-AIY

anti-phase-like activities observed in starved animals at higher temperature can explain the navigation toward colder region, since AIY calcium signals were increased with cooling and decreased with warming (Fig. 4G(d)). However, isothermal tracking might require

highly-regulated sensory-motor coupling by crosstalk between upstream and downstream neurons. In particular, worms need to integrate their head swing, position and temporal thermal stimuli in a certain time window and fine-tune their behavior to remain at around the cultivation temperature. C. elegans is thought to execute sensory-motor integration during crawling along isotherms on a thermal gradient, and such a sensorimotor control can be computed by RIA interneurons (Hendricks, 2012; Liu et al., 2018; Ouellette et al., 2018), a postsynaptic partner of AIY (White et al., 1986). We could not explain the behavior solely by AFD-AIY activities (Fig. 4G (c)), probably due to a lack of insights to the activity patterns of further downstream neurons in the thermotaxis circuit (Ikeda et al., 2018; Mori and Ohshima, 1995). Simultaneous calcium imaging for AFD, AIY and RIA and other neurons will uncover a neural logic for the isothermal tracking that allows animals to remain at around cultivation temperature.” (lines 487-511)

Q3. Finally, I liked the authors’ hypothesis that the phase shift differences could be the result of varying the strengths of inhibitory and excitatory transmission from AFD to AIY with different temporal dynamics. Is there any molecular evidence in support of this idea, or experiments that could test this hypothesis?

A3. Our short answer is “We do not know the details at this stage", but there are two molecules that might be involved in the excitatory-inhibitory interaction. AFD is known to communicate with AIY via both neuropeptide and glutamate (Narayan et al., 2011; Ohnishi et al., 2011). The peptidergic synaptic transmission is excitatory signal (Narayan et al., 2011), on the other hand, the glutamatergic synaptic transmission is inhibitory signal (Ohnishi et al., 2011). The previous study showed that UNC-31, C. elegans ortholog of calcium-dependent activator protein required for the secretion of neuropeptide (Speese et al., 2007) and EAT-4, C. elegans homolog of

sodium-dependent inorganic phosphate cotransporter required for glutamatergic transmission (Lee et al., 1999) function in AFD to regulate the excitatory-inhibitory balance onto AIY (Nakano et al., 2019). The opposing signals from AFD via UNC-31 and EAT-4 might generate various patterns of postsynaptic response in AIY as we theoretically predicted above. Regulating the threshold for neurotransmitter release and the rate of recovery or degradation might control the timing and sharpness of these opposing signals. (lines 536-548)

Finally, we corrected some typos (lines 239-240, 819, 853, 878-879, 882-883, 888, 890, 904-906 and 910-912).

Again, thank you for giving us the opportunity to improve our manuscript with your thoughtful comments and queries. We hope that you will find our revised manuscript suitable for publication in eNeuro.

Sincerely yours, Authors
